# The coupling of global brain activity and cerebrospinal fluid flow as a potential predictive marker of brain amyloid-β accumulation

**DOI:** 10.1016/j.tjpad.2025.100228

**Published:** 2025-06-13

**Authors:** Yuya Tanaka, Koji Kamagata, Yuya Saito, Kaito Takabayashi, Rinako Iseki, Wataru Uchida, Christina Andica, Akifumi Hagiwara, Akihiko Wada, Toshiaki Akashi, Osamu Abe, Shigeki Aoki

**Affiliations:** a Department of Radiology, Juntendo University Graduate School of Medicine, 2-1-1 Hongo, Bunkyo, Tokyo 113-8421, Japan; b Department of Radiology, Graduate School of Medicine, The University of Tokyo, 7-3-1 Hongo, Bunkyo, Tokyo 113-8655, Japan; c Faculty of Health Data Science, Juntendo University, 6-8-1 Hinode, Urayasu, Chiba 279-0013, Japan

**Keywords:** Alzheimer’s disease, Amyloid-β, Cerebrospinal fluid clearance, Glymphatic system, Resting-state functional MRI, gBOLD–CSF coupling

## Abstract

**Background:**

Impaired cerebrospinal fluid (CSF) clearance is thought to contribute to amyloid-β (Aβ) accumulation in Alzheimer’s disease (AD). Global brain activity–CSF flow coupling (gBOLD–CSF coupling), measured through resting-state functional MRI, reflects CSF clearance capacity. A higher coupling value indicates weaker coupling. Its potential as a predictive marker for Aβ accumulation remains unclear.

**Objectives:**

This study aims to determine whether weaker gBOLD–CSF coupling precedes Aβ accumulation in cognitively normal, Aβ-negative individuals and to explore its predictive potential for amyloid conversion.

**Design:**

A longitudinal observational study using Alzheimer’s Disease Neuroimaging Initiative (ADNI) data.

**Setting:**

Data from ADNI-participating sites.

**Participants:**

16 cognitively normal participants, initially Aβ-negative: seven fast-converters (transitioned to Aβ-positive within two years) and nine slow-converters (remained Aβ-negative for at least two years).

**Measurements:**

gBOLD–CSF coupling was calculated as the Pearson correlation coefficient between global Blood-Oxygen-Level-Dependent (BOLD) and CSF inflow signals. Group differences in gBOLD–CSF coupling were analyzed, along with partial correlation analyses between gBOLD–CSF coupling and annual changes in Aβ biomarkers and cognitive scores.

**Results:**

Fast-converters showed significantly higher gBOLD–CSF coupling values, indicating weaker coupling (Cohen’s *d =* 1.76, *p =* 0.012). Coupling values positively correlated with annual changes in Aβ-PET SUVR (*r =* 0.594, *p =* 0.054) and negatively with MoCA scores (*r =* −0.654, *p =* 0.021).

**Conclusion:**

Weaker gBOLD–CSF coupling precedes brain Aβ accumulation, indicating its potential as a predictive marker for amyloid conversion. Future studies should refine clinical thresholds for early intervention strategies in AD prevention.

## 1 Introduction

Accumulating evidence suggests that amyloid-β (Aβ) deposition in the brain associated with Alzheimer’s disease (AD), particularly in the most common type—late-onset AD (LOAD)—results from impaired clearance mechanisms such as the glymphatic system [[1], [2], [3]]. However, definitive studies using human clinical data to directly demonstrate this sequence of events are still forthcoming, leaving the causality between Aβ accumulation and impaired clearance unresolved. It is hypothesized that some patients exhibit reduced clearance capacity and are expected to develop Aβ brain accumulation in the near future, even if they currently show no evidence of Aβ deposition and have normal cognitive function. These patients, anticipated to convert from Aβ-negative (A−) to Aβ-positive (A+) in the near future, will be referred to as **“**fast-converters,**”** based on previous research [4]. Identifying fast-converters using a measure of clearance could provide evidence that reduced clearance precedes and may cause Aβ accumulation. This, in turn, could highlight an upstream and fundamental target for AD prevention strategies.

With regard to the clearance mechanism mentioned above, cerebrospinal fluid (CSF) clearance plays a critical role in maintaining brain health, particularly through the glymphatic system that is significantly enhanced during non-rapid eye movement (NREM) sleep [5,6]. This system is an intracranial clearance pathway that utilizes unique perivascular channels [[5], [6], [7]], primarily formed by astroglial cells [[5], [6], [7], [8], [9]], to remove metabolic waste such as Aβ and tau from the interstitial space (ISF) of the brain parenchyma (BP) [[5], [6], [7], [8], [9], [10]]. The pathway involves the bulk flow of CSF along perivascular spaces (PVS) surrounding arteries [[5], [6], [7], [8], [9], [10]]. This CSF then exchanges with the ISF within the BP via aquaporin-4 (AQP4) water channels predominantly located in the astrocytic endfeet, effectively **“**flushing**”** the tissue and collecting soluble waste products [[5], [6], [7], [8], [9], [10]]. Subsequently, CSF and entrained waste egress via perivenous spaces [[5], [6], [7], [8], [9], [10]]. Impairment or dysfunction of the glymphatic system is therefore hypothesized to contribute to the accumulation of these toxic proteins, potentially driving the initiation and progression of various neurological disorders, such as Alzheimer’s disease (AD) [11,12] and Parkinson’s disease (PD) [13].

Evaluating glymphatic function in living humans using direct methods can be challenging. Recent research has suggested that the coupling strength between the global blood-oxygen-level-dependent (gBOLD) signal and CSF inflow dynamics, measured non-invasively using resting-state functional magnetic resonance imaging (rsfMRI), can serve as a potential indicator of glymphatic function [14]. The gBOLD signal reflects large-scale neural and physiological modulations, specifically low-frequency oscillations (<0.1 Hz) that are linked to transient arousal states and are significantly stronger during sleep or drowsiness [14,15]. These large-scale gBOLD fluctuations are associated with some physiological reactions via sympathetic activations such as arterial pulsations [16,17], spontaneous changes in vessel tone [17] and possibly AQP4 activation mostly located on endfeet of astrocytes [18]. These physiological changes are hypothesized to be the driving forces behind CSF flow along the perivascular spaces. gBOLD–CSF coupling, calculated as the correlation coefficient between the gBOLD signal and the CSF inflow signal around the medulla [15,[18], [19], [20], [21]], is typically a negative correlation: peaks in CSF inflow arise during the rapid decline of the gBOLD signal—that is, at the positive-to-negative turning point—and therefore lag the gBOLD signal [14]. This relationship is consistent with the Monro–Kellie doctrine, which suggests that a decrease in cerebral blood volume leads to an increase in CSF inflow to maintain constant intracranial volume [14,22]. Therefore, gBOLD–CSF coupling is increasingly understood as an integrated reflection of the coordinated activity of these neural and physiological processes that are tightly linked to glymphatic clearance [18].

Importantly, altered gBOLD–CSF coupling has been associated with clinical manifestations of various neurological disorders. Studies have shown that reduced gBOLD–CSF coupling is associated with the severity of small vessel disease (SVD) [20]. In Parkinson’s disease (PD), gBOLD–CSF coupling has been found to be weaker in drug-naïve patients compared to healthy controls [21], and is linked to cognitive impairment [19] and longitudinal motor impairment [21].

Several studies have investigated the relationship between AD and gBOLD–CSF coupling. One study reported that gBOLD–CSF coupling weakened as subjects progressed through the stages of **“**healthy control**”** (HC), **“**significant memory concern**”** (SMC), **“**mild cognitive impairment**”** (MCI), and **“**AD**”** [15]. Another study demonstrated that A+ subjects exhibited weaker gBOLD–CSF coupling compared to A− subjects, with a significant negative correlation between gBOLD–CSF coupling and two-year changes in the standardized uptake value ratio (SUVR) of Aβ-PET ([^18^F]florbetapir) across the entire cortex (Aβ-PET SUVR) [18]. However, it remains unclear whether a decrease in gBOLD–CSF coupling is a cause or a consequence of Aβ accumulation, as those studies may not place much focus on the **“**AD continuum**”** principle [23], which describes AD as a continuous process beginning with Aβ deposition, followed by Tau-mediated brain damage, progressing through mild cognitive impairment (MCI) due to AD, and ultimately culminating in AD dementia. To explore this causality, longitudinal studies with attention to the **“**AD continuum**”** principle are necessary to determine whether reduced CSF clearance precedes Aβ accumulation.

Therefore, this study hypothesizes that reduced gBOLD–CSF coupling, reflecting reduced CSF clearance precedes Aβ accumulation in the brain. The objective is to demonstrate that gBOLD–CSF coupling may serve as a predictive marker for brain Aβ accumulation in A− and cognitively normal individuals. Specifically, the following longitudinal analysis was conducted in this study. Among cognitively normal and A− subjects, **“**fast-converters**”** who would become A+ in the near future (particularly within two years) were identified and compared with **“**slow-converters,**”** who would eventually become A+ but not within two years, focusing on differences in gBOLD–CSF coupling between these groups. Additionally, correlations were analyzed between annual changes in gBOLD–CSF coupling and Aβ biomarker values (and cognitive scores) during the period leading up to conversion to A+, prior to the preclinical AD stage.

## 2 Methods

### 2.1 Participants and study data

The data analyzed in this investigation were sourced from the Alzheimer’s Disease Neuroimaging Initiative (ADNI) database [24]. Established in 2003 as a collaborative effort between public and private sectors, under the guidance of Principal Investigator Michael W. Weiner, MD, the primary aim of ADNI is to assess the utility of integrating serial MRI, positron emission tomography (PET), additional biomarkers, and clinical and neuropsychological evaluations in monitoring the advancement of MCI and early-stage AD. Prior to inclusion in the ADNI database, all participants provided written informed consent for the storage and use of their clinical and imaging data for research purposes. Ethical oversight was ensured through the acquisition of approval from the institutional review board (IRB) at each ADNI participating site (detailed protocol: http://adni.loni.usc.edu/wp-content/uploads/2013/09/DOD-ADNI-IRB-Approved-Final-protocol-08072012.pdf). Data acquisition within ADNI was performed in compliance with the ethical standards articulated in the Declaration of Helsinki. Current information regarding the initiative can be found at www.adni-info.org. Utilization of the ADNI database in this study was conducted in full adherence to the stipulated ADNI data use agreements.

The dataset utilized comprised the ADNI-GO, ADNI-2, and ADNI-3 data (version: 2023-08-21, URL: http://adni.loni.usc.edu/). This dataset includes data on 2,430 individuals, encompassing variables such as age, sex, APOE gene genotypes, years of education, longitudinal diagnoses (cognitively normal [CN], MCI, or AD), cognitive function scores (e.g., MMSE), CSF levels of Aβ42, SUVR data of ^18^F-AV45 (Florbetapir; FBP) amyloid PET, and MRI images including rsfMRI. Initially, the dataset was reduced to 1,123 subjects for whom rsfMRI data were available. Subsequently, data from the period during which participants were CN were extracted, resulting in a dataset encompassing information from 586 individuals. Additionally, based on the definition and concept of the **“**AD continuum**”**, we excluded those who were potentially unrelated to Aβ pathology, i.e., those who never reached A+ status during the follow-up period. This left 165 individuals who had exhibited A+ status at least once. The criteria for A+ status were defined as CSF Aβ42 levels of ≤ 880 pg/mL [25,26] or global SUVRs of FBP ≥ 1.11 [[26], [27], [28], [29]], based on previous studies. Among these 165 subjects, 122 who were already A+ at baseline were excluded, leaving 43 who were A− status at baseline. From this population, two age-matched groups were defined: (1) **“**fast-converter**”** group (*n* = 7), who converted from A− to A+ within two years of rsfMRI, and (2) **“**slow-converter**”** group (*n* = 9), who remained A− for at least two years. This two-year follow-up period was determined in accordance with previous studies [15,18]. The length of **“**two years**”** was defined as 24 months according to the Visit code. For the combined total of 16 patients in the aforementioned two groups, the actual measurement of the length of the **“**two years**”** follow-up period was 2.0 ± 0.1 years.

### 2.2 Image acquisition and preprocessing

All rsfMRI data were acquired using 3 Tesla MRI scanners at various ADNI participating sites following a standardized protocol (https://adni.loni.usc.edu/data-samples/adni-data/neuroimaging/mri/mri-scanner-protocols/). The scans were conducted on recent models of MRI scanners from General Electric Healthcare (Illinois, USA), Philips Medical Systems (Amsterdam, Netherlands), and Siemens Medical Solutions (Erlangen, Germany). Each imaging session commenced with a high-resolution T1-weighted MPRAGE sequence (TR/TE = 2300/3.1 ms) for anatomical reference and normalization. The rsfMRI acquisition involved 140 (ADNI-GO and ADNI-2) or 200 (ADNI-2 extended fMRI sessions and ADNI-3) functional volumes, utilizing a gradient-echo echo-planar imaging (EPI) sequence. Scanning parameters included a flip angle of 80° (ADNI-GO and ADNI-2) or 90° (ADNI-3), spatial resolution of 3 × 3 × 3 mm³, and slice thickness of 3.3 mm (ADNI-GO and ADNI-2) or 3.4 mm (ADNI-3), with TR/TE = 3000/30 ms. To preprocess rsfMRI, the typical pipeline in CONN version 21a based on previous studies was employed. Key steps included motion correction, skull stripping, spatial smoothing (full width at half maximum [FWHM] = 4 mm), bandpass filtering (0.01–0.1 Hz), and removal of linear and quadratic trends. To ensure steady-state magnetization and mitigate temporal filtering edge effects, the first and last 5 volumes of each rsfMRI session were discarded [15,18]. Subsequently, co-registration of the preprocessed fMRI data to the corresponding T1-weighted image was performed [30], followed by transformation to the 152-brain Montreal Neurological Institute (MNI-152) standard space [31], also using CONN version 21a. The data were resampled to a spatial resolution of 3 × 3 × 3 mm³. The global signal, CSF signal, and motion parameter regressions were omitted to preserve the global BOLD signal integrity, crucial for gBOLD–CSF coupling analysis [32].

Amyloid PET data were acquired approximately 50–70 min post-injection of FBP, following an established ADNI protocol (https://adni.loni.usc.edu/wp-content/uploads/2010/05/ADNI2_PET_Tech_Manual_0142011.pdf). The amyloid PET images underwent averaging, spatial alignment, interpolation to a standard voxel size (1.5 × 1.5 × 1.5 mm³), spatial smoothing (FWHM = 8 mm), and registration to T1-weighted MRI space. Cortical Aβ SUVR was calculated as the ratio of the mean florbetapir uptake in gray matter to the composite reference region, encompassing the entire cerebellum, brainstem/pons, and subcortical white matter [33]. These SUVR data were included in the aforementioned dataset utilized in this study.

### 2.3 gBOLD–CSF coupling calculation

**Fig. 1:**
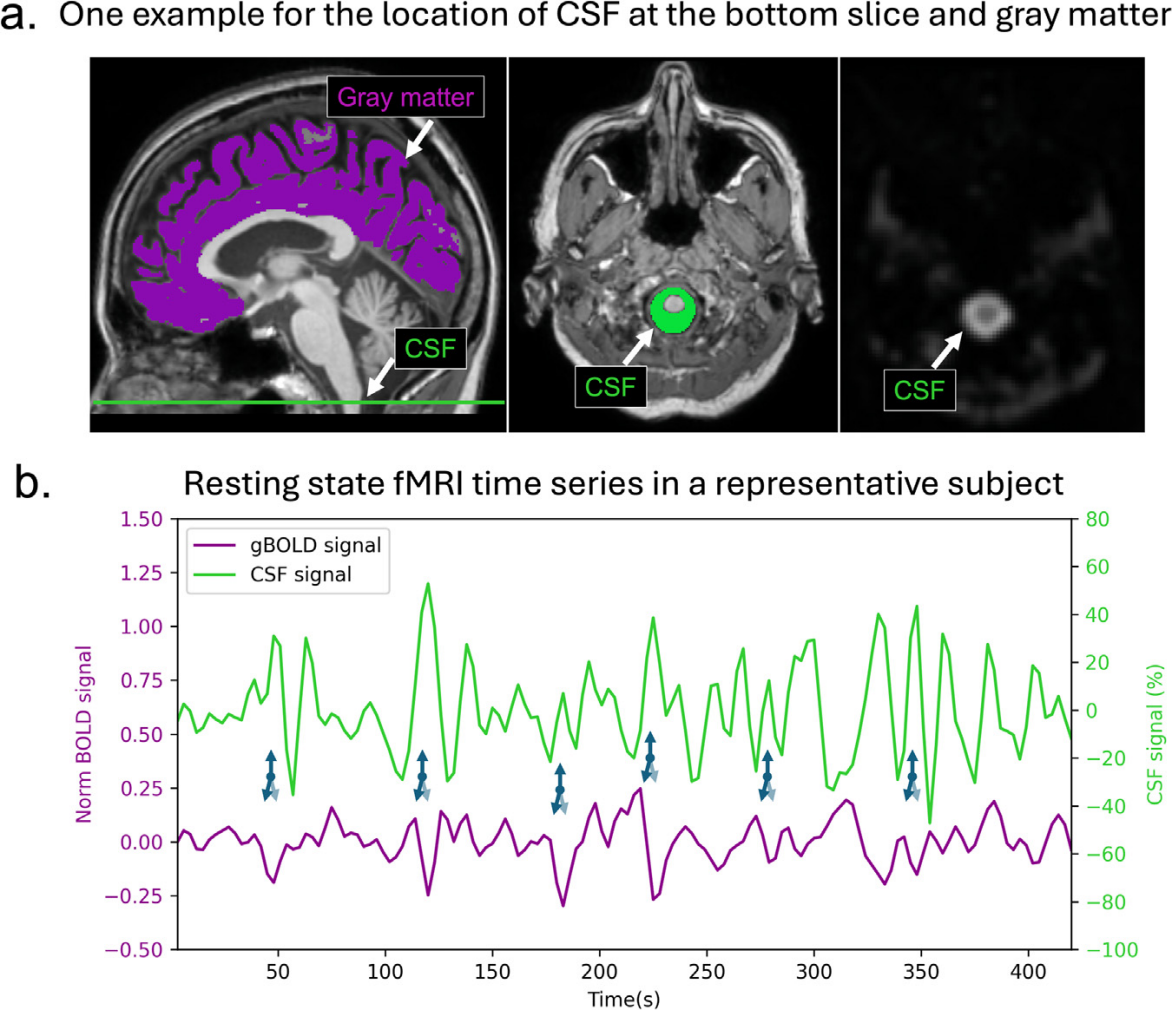
Correlation between global BOLD signal and CSF dynamics in fMRI measurements. a, The global Blood-Oxygen-Level-Dependent (gBOLD) signal analysis was performed on gray matter regions, as visualized by the purple overlay on the structural T1-weighted MRI (left panel). CSF signal measurements were obtained from the designated CSF regions located at the inferior-most slice of the fMRI acquisition volume around the medulla (displayed in green mask, middle panel). The CSF region exhibits a distinctly higher intensity compared to adjacent tissues in T2*-weighted images (right panel). b, Representative time course data from a single participant demonstrates temporal synchronization between gBOLD and CSF signals. Peaks in CSF inflow occur during the rapid decrease of the gBOLD signal, i.e., during the positive-to-negative peak of the gBOLD signal and thus lag behind the gBOLD signal. The temporal association between these signals suggests a systematic coupling mechanism in brain physiology. CSF, cerebrospinal fluid.

To quantify gBOLD–CSF coupling, the gBOLD and CSF signals were first extracted from the rsfMRI data. The gBOLD signal, representing global brain activity, was calculated by averaging the BOLD signal across all gray matter voxels (Fig. 1a) [34]. Gray matter masks were defined based on the Harvard–Oxford cortical and subcortical structural atlases [31]. The CSF signal, reflecting CSF inflow [35], was extracted from the bottom slice of the fMRI acquisition around the medulla, as this slice is most sensitive to inflow effects (Fig. 1a) [14]. Major changes in the global BOLD signal were commonly accompanied by corresponding major changes in the CSF signal, suggesting a coupling of the two, as indicated by previous studies (Fig. 1b) [14,15,[18], [19], [20], [21]]. gBOLD–CSF coupling was quantified using the cross-correlation function between the preprocessed gBOLD and CSF signals. The representative value of coupling was defined as the Pearson correlation coefficient at a time lag of +3 s, in accordance with previous research [15,18].

### 2.4 Statistical analysis

Differences between fast-converters and slow-converters were assessed using two-sample t-tests for continuous variables (age, years of education, and cognitive function scales) and chi-square tests for categorical variables (sex, the presence or absence of the APOE ε4 allele [−/+], and ADNI phase).

The difference in gBOLD–CSF coupling between fast-converters and slow-converters was compared using a generalized linear model, adjusting for age, sex, APOE ε4 allele presence/absence, and years of education. An additional analysis was also performed by adding ADNI phase to the covariates. Effect size was calculated as Cohen’s *d*. Furthermore, the relationship between gBOLD–CSF coupling and the annual rate of change (from the time of the gBOLD–CSF coupling quantification) in Aβ biomarkers (Aβ-PET SUVR and CSF levels of Aβ42) and cognitive function scales, such as Mini-Mental State Examination (MMSE) and Montreal Cognitive Assessment (MoCA), was evaluated using partial correlation analysis, adjusting for age, sex, APOE ε4 allele presence/absence, and years of education. These analyses were conducted for all participants as well as separately for the fast-converter and slow-converter groups. Additional analyses were also performed by adding ADNI phase to the covariates.

A p-value of < 0.05 was considered statistically significant (*). Correlation coefficients were interpreted according to the following guidelines: |r| < 0.20 was considered very weak, 0.20–0.39 weak, 0.40–0.59 moderate, 0.60–0.79 strong, and 0.80–1.00 very strong correlation. All statistical analyses were performed using SPSS version 29.0 (IBM Corp., Armonk, NY, USA).

## 3 Results

### 3.1 Participant characteristics

The imaging and clinical data from 16 participants of the ADNI project were utilized. This population consisted of individuals who remained cognitively normal (CN) throughout the follow-up period and were identified as A− at the initial visit but were subsequently found to be A+ at any point during the follow-up period. Among these participants, seven were classified into the “fast-converter” group, having converted from A− to A+ within two years following the rsfMRI, and nine confirmed to have remained A− for at least two years were classified into the “slow-converter” group. Table 1 summarizes the participant characteristics, including age, sex, APOE ε4 allele presence/absence, years of education, ADNI phase, and cognitive function scales. No significant differences were observed in any examined characteristics between fast-converters and slow-converters.

**Table 1:** Demographic characteristics of study participants.

Total *n* = 16	Fast-converters (*n* = 7)	Slow-converters (*n* = 9)	p-value
Age	78.75 ± 4.75	78.24 ± 7.74	0.873
Sex (M: F)	3: 4	1: 8	0.146
APOE ε4 allele (−: +)	5: 2	5: 4	0.515
Years of education	17.29 ± 1.89	17.56 ± 2.19	0.799
ADNI phase (GO/2/3)	0/2/5	1/2/6	0.652
MMSE scores	27.86 ± 1.35	29.11 ± 0.78	0.055
MoCA scores	26.71 ± 1.70	26.33 ± 2.12	0.696
FAQ scores	0.14 ± 0.38	0.56 ± 1.67	0.490
CDR-SB scores	0.21 ± 0.27	0.00 ± 0.00	0.078
RAVLT-immediate scores	46.43 ± 14.86	49.22 ± 8.89	0.670
RAVLT-learning scores	6.14 ± 2.41	5.44 ± 2.60	0.588
RAVLT-forgetting scores	2.86 ± 2.67	3.56 ± 4.25	0.694
RAVLT-% forgetting scores	26.68 ± 25.75	34.45 ± 38.96	0.640
ADAS-11 score	4.86 ± 2.83	5.15 ± 2.30	0.829
ADAS-13 score	8.00 ± 3.91	7.93 ± 3.49	0.969
ADAS-Q4 score	2.86 ± 1.57	2.33 ± 1.66	0.530
LDELTOTAL	14.00 ± 3.70	14.56 ± 2.88	0.749
TRABSCOR	67.57 ± 16.03	62.56 ± 17.81	0.564

Numerical data are expressed as mean ± 1 SD (standard deviation). The difference between the two groups was tested using the chi-square test for patient sex, the presence or absence of APOEε4 allele [−/+], and ADNI phase, whereas a two-sample t-test was used for other parameters. No significant differences were found between the two groups for all characteristics.

ADAS, Alzheimer’s Disease Assessment Scale; CDR-SB, Clinical Dementia Rating Scale Sum of Boxes; FAQ, Functional Activities Questionnaire; LDELTOTAL, Logical Memory Delayed Recall Total Score; MF, male/female; MMSE, Mini-Mental State Examination; MoCA, Montreal Cognitive Assessment; RAVLT, Rey Auditory Verbal Learning Test; TRABSCOR, time to complete Trail Making Test Part B.

### 3.2 Comparison of the gBOLD–CSF coupling between fast-converters and slow-converters

**Fig. 2:**
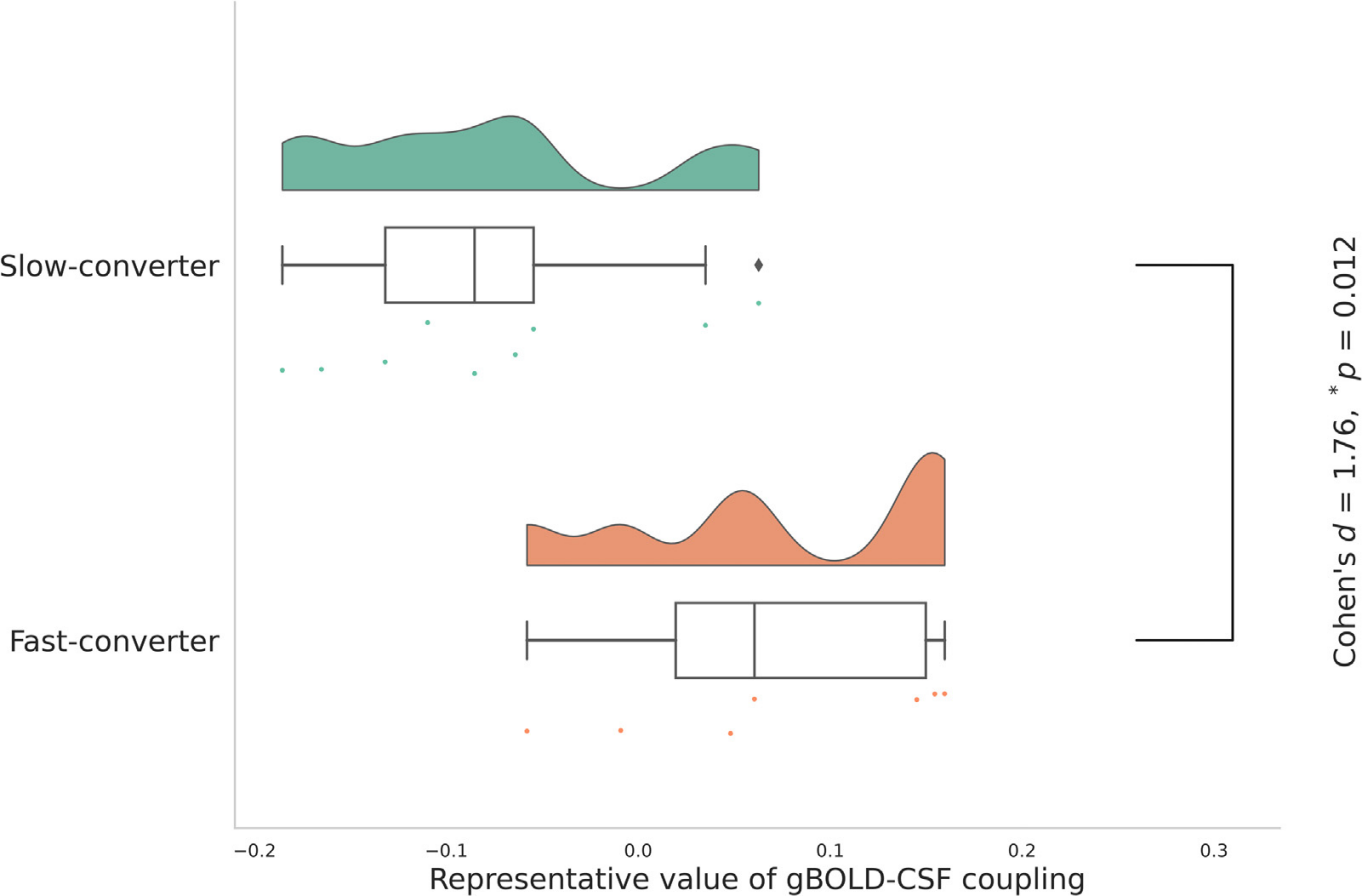
Comparison of the gBOLD–CSF coupling between fast-converters and slow-converters. The representative value of gBOLD–CSF coupling was significantly high in fast-converters compared to slow-converters (Cohen’s *d* = 1.76, *p = 0.012). Note that the higher the representative value of gBOLD–CSF coupling, the weaker the gBOLD–CSF coupling. The analysis was performed with adjustments for age, sex, APOE ε4 allele presence/absence, and years of education. gBOLD, global Blood-Oxygen-Level-Dependent signals; CSF, cerebrospinal fluid.

A generalized linear model was employed to compare the gBOLD–CSF coupling between fast-converters (*n* = 7) and slow-converters (*n* = 9), controlling for age, sex, APOE ε4 allele presence/absence, and years of education as covariates. An additional analysis was also performed by adding ADNI phase to the covariates. The representative value of gBOLD–CSF coupling was significantly higher (i.e., weaker gBOLD–CSF coupling) in fast-converters compared to slow-converters (Cohen’s *d* = 1.76; *p = 0.012; Fig. 2, [an additional analysis] *p = 0.019; Supplementary Fig. 2).

### 3.3 Correlation between gBOLD–CSF coupling and Aβ biomarkers and cognitive function scales

**Fig. 3:**
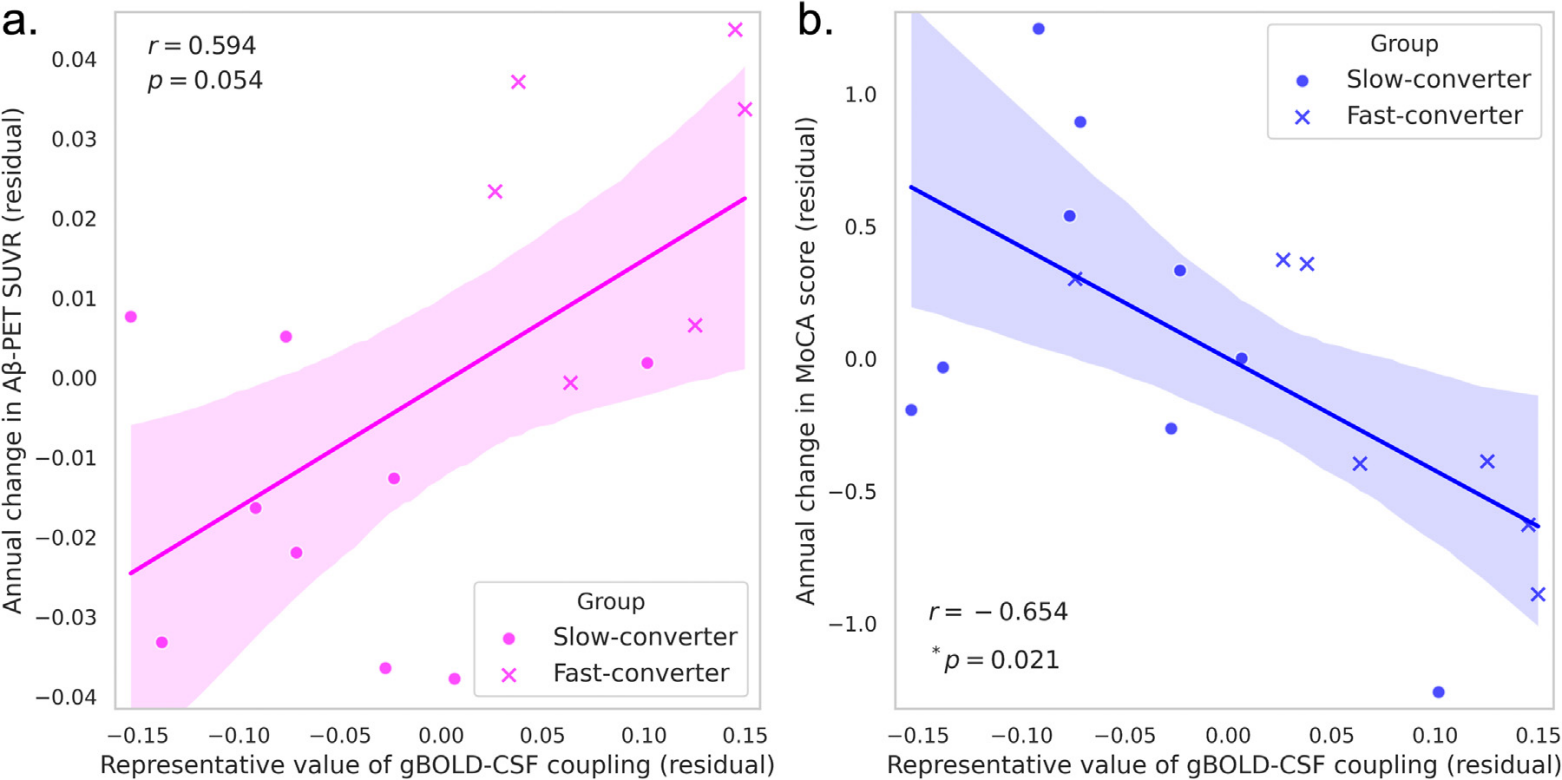
Partial correlation between gBOLD–CSF coupling representative value and annual Aβ-PET SUVR and MoCA score change. a, Partial correlation analysis between the representative value of gBOLD–CSF coupling and the annual change in Aβ-PET SUVR revealed a moderate positive correlation (*r* = 0.594, *p* = 0.054). b, The partial correlation analysis between the representative value of gBOLD–CSF coupling and the annual change in MoCA score showed a significant strong negative correlation (*r* = −0.654, *p = 0.021). Both plots are adjusted for the following covariates: age, sex, APOE ε4 allele presence/absence, and years of education. The residuals are displayed as separate data points. Note: Higher representative values of gBOLD–CSF coupling indicate weaker coupling. Aβ, amyloid beta; PET, positron emission tomography; SUVR, standardized uptake value ratio; MoCA, Montreal Cognitive Assessment.

The relationship between gBOLD–CSF coupling and annual changes from the time of gBOLD–CSF coupling quantification in Aβ biomarkers (Aβ-PET SUVR and CSF levels of Aβ42) and cognitive function scales, such as MMSE and MoCA, was evaluated for all participants (*n* = 16), as well as separately for the fast-converter group (*n* = 7) and the slow-converter group (*n* = 9), using partial correlation analysis while controlling for age, sex, APOE ε4 allele presence/absence, and years of education. A partial correlation analysis between the representative value of gBOLD–CSF coupling and the annual change in Aβ-PET SUVR in all participants (*n* = 15, due to missing values for one participant) revealed a moderate positive correlation (*r* = 0.594; *p* = 0.054, Fig. 3a). A similar analysis with ADNI phase added as a covariate showed a strong positive correlation (*r* = 0.604; *p* = 0.064, Supplementary Fig. 3a). A partial correlation analysis for the annual change in CSF levels of Aβ42 was not conducted due to data availability for only one participant. Furthermore, partial correlation analyses between the representative value of gBOLD–CSF coupling and cognitive function scales in all participants demonstrated a significant strong negative correlation with the annual change in MoCA score (*r* = −0.654; *p = 0.021, Fig. 3b). Similar analyses with ADNI phase added as a covariate also showed a strong negative correlation (*r* = −0.642; *p = 0.033, Supplementary Fig. 3b). The partial correlation analyses within the slow-converter group showed no significant correlation for any indicators, including Aβ biomarkers and cognitive function scales. The same analysis could not be performed within the fast-converter group due to insufficient degrees of freedom (*df* = 0) resulting from the small sample size.

## 4 Discussion

This study sought to clarify the relationship between Aβ accumulation and CSF clearance prior to preclinical AD by comparing the gBOLD–CSF coupling—an increasingly reported indicator of the glymphatic system, which is thought to be the primary mechanism for clearing toxic wastes in the brain such as Aβ and tau—between fast-converters and slow-converters. The findings indicated that gBOLD–CSF coupling was weaker in fast-converters than in slow-converters. Additionally, correlations between gBOLD–CSF coupling and Aβ biomarkers and cognitive function scales were examined, revealing a moderate correlation or a strong inverse correlation between gBOLD–CSF coupling and the annual change in Aβ-PET SUVR and MoCA score, respectively. Thus, to our knowledge, this is the first longitudinal study demonstrating the relationship between fast-converters and CSF clearance, suggesting that reduced CSF clearance may lead to brain Aβ accumulation.

In prior studies examining the relationship between AD and gBOLD–CSF coupling [15,18], researchers did not confirm that the subjects were encompassed within the “AD continuum” as rigorously as in the current study. Instead, they categorized participants as HC, SMC, MCI, and AD based on clinical stages of Alzheimer’s disease, reporting that gBOLD–CSF coupling weakened in a stepwise manner across these categories [15]. It is postulated that some subjects classified as “HC” may have included individuals who would subsequently convert to A+. From the perspective of early detection and intervention, the ability to identify fast-converters holds great significance. To investigate this discriminability, a comparison of CSF clearance between fast-converters and slow-converters using gBOLD–CSF coupling was performed. This is based on the recent hypothesis that the primary cause of A+ status is impairment of CSF clearance [[1], [2], [3]]. The criteria for defining fast-converters were stringently applied, as were the criteria for slow-converters. Specifically, elderly participants who were CN and Aβ-negative (A−) at baseline but eventually became A+ were divided into two groups: those who remained CN but converted to A+ within two years (fast-converters) and those who remained A− for at least two years (slow-converters). This examination demonstrated that fast-converters exhibited weaker gBOLD–CSF coupling compared to slow-converters, indicating that differentiating fast-converters from slow-converters is feasible based on gBOLD–CSF coupling. Additionally, findings suggest that reduced CSF clearance precedes amyloid conversion.

Moreover, to provide further insight into a causal relationship between declining clearance and Aβ accumulation prior to preclinical AD, the correlation between gBOLD–CSF coupling and annual changes in Aβ-PET SUVR was examined. A moderate inverse correlation was identified between these two factors. These findings, in conjunction with earlier observations that decreased CSF clearance precedes amyloid conversion, suggest a potential link between decreased CSF clearance and subsequent amyloid accumulation. Two previous studies examined the correlation between gBOLD–CSF coupling and two-year changes in Aβ-PET SUVR [15,18]. One study found no significant correlation within a group comprising **“**HC,**” “**SMC,**” “**MCI,**”** and **“**AD” [15]. Another study reported a significant inverse correlation in patients categorized as **“**HC,**” “**SMC,**”** or **“**MCI,**”** who were confirmed A+ based on CSF levels of Aβ42 but not Aβ-PET [18]. It is known that Aβ accumulation approaches a plateau between “MCI” and “AD” [23]. Therefore, the difficulty is highlighted in discerning an inverse correlation between gBOLD–CSF coupling and the rate of Aβ accumulation in subjects exhibiting marked and approaching plateau levels of Aβ in the brain. In the current study, which included only subjects in the earliest stages of Aβ accumulation, there was a moderate and not statistically significant correlation (*r* = 0.594; *p* = 0.054). As will be discussed later, the current study has the limitation of the small sample size due to the very strict inclusion criteria. Further studies with a larger sample size and greater statistical power are needed to clarify the relationship between gBOLD–CSF coupling and annual changes in Aβ-PET SUVR in patients in the earliest stages of Aβ accumulation.

In the concurrent analysis of the relationship between gBOLD–CSF coupling and annual changes in MoCA scores, a strong correlation was discovered. The patients included in this study were in a stage preceding preclinical AD on the “AD continuum,**”** rendering the explanation of this result in the context of the “AD continuum” alone complicated. Conversely, decreased clearance can negatively impact cognitive function by inducing neuroinflammation [36,37], and this mechanism may be implicated in the observed results. gBOLD–CSF coupling may also hold potential for future studies regarding such pathological conditions.

This study has two limitations. First, the mechanisms underlying gBOLD–CSF coupling remain unclear due to the absence of validation through autopsy studies, although gBOLD–CSF coupling represents a noteworthy potential indicator that can be obtained non-invasively via rsfMRI for evaluating the glymphatic system. The prevailing hypothesis is that the repetitive fluctuations in cerebral blood volume, mediated by the slow global brain activity that is prominent during sleep and drowsiness, promote CSF flow due to the rule that intracranial volume is kept constant (Monro–Kellie doctrine) [14,20]. Consequently, the existence of intervening factors that determine the smoothness of this coupling should be assumed, and the sum of these interindividual differences may be responsible for the differences in gBOLD–CSF coupling. Indeed, the gBOLD signal has recently been considered in relation to AQP4 activation, which is mainly localized on astrocytic endfeet[18]. Given these considerations, gBOLD–CSF coupling may be better interpreted as an integrative index reflecting the components of the glymphatic system, which may be why we were able to point out the difference between fast-converters and slow-converters in the present study; however, further basic research utilizing animal models is essential to attain a comprehensive understanding of this phenomenon. The second limitation was the small sample size. In general, a small sample size increases the relative impact of noise and decreases statistical power. And, specifically in this study, the small sample size may have affected the interpretation of the results, since a relatively high proportion of positive high coupling values, which might have been treated as outliers or noise if they were in a large sample, were not excluded in this study. Future research is needed to interpret the significance of positive high coupling values, whether they should be excluded as outliers or reflect a disruption or alteration in coupling. The small sample size also prevented calculation of a reliable cutoff value for gBOLD–CSF coupling that could distinguish between fast-converters and slow-converters. This limitation poses a challenge for direct clinical application. Future research will aim to determine the cut-off value for gBOLD–CSF coupling with a view to direct clinical application using a larger cohort.

## 5 Conclusion

In conclusion, this study provides initial evidence that reduced CSF clearance, as measured by gBOLD–CSF coupling—an innovative marker for the glymphatic system—precedes and may instigate brain Aβ accumulation in CN elderly populations. gBOLD–CSF coupling holds potential to predict future Aβ accumulation in the brains of CN elderly individuals, potentially enabling earlier medical interventions for AD prevention. Future investigations should concentrate on elucidating the underlying mechanisms, and enhancing clinical applicability.

## CRediT authorship contribution statement

**Yuya Tanaka:** Writing – review & editing, Writing – original draft, Visualization, Validation, Supervision, Software, Resources, Project administration, Methodology, Investigation, Funding acquisition, Formal analysis, Data curation, Conceptualization. **Koji Kamagata:** Writing – review & editing, Validation, Supervision, Resources, Project administration, Methodology, Investigation, Funding acquisition, Formal analysis, Data curation, Conceptualization. **Yuya Saito:** Software, Formal analysis, Data curation. **Kaito Takabayashi:** Visualization, Formal analysis, Data curation. **Rinako Iseki:** Visualization, Formal analysis. **Wataru Uchida:** Software, Formal analysis. **Christina Andica:** Validation. **Akifumi Hagiwara:** Writing – review & editing, Validation. **Akihiko Wada:** Writing – review & editing, Validation. **Toshiaki Akashi:** Writing – review & editing, Validation. **Osamu Abe:** Writing – review & editing, Validation. **Shigeki Aoki:** Writing – review & editing, Validation, Supervision.

## Declaration of interests

The authors declare the following financial interests/personal relationships which may be considered as potential competing interests:

Koji Kamagata reports financial support was provided by Japan Society for the Promotion of Science. Yuya Tanaka reports financial support was provided by Japan Society for the Promotion of Science. Koji Kamagata reports financial support was provided by Japan Science and Technology Agency. Koji Kamagata reports financial support was provided by Japan Agency for Medical Research and Development. If there are other authors, they declare that they have no known competing financial interests or personal relationships that could have appeared to influence the work reported in this paper.

## Appendix Supplementary materials

Image, application 1
