# Supplementary material for: The coupling of global brain activity and cerebrospinal fluid flow as a potential predictive marker of brain amyloid-β accumulation
Source: J Prev Alzheimers Dis. 2025 Jun 13;12(8):100228. doi: 10.1016/j.tjpad.2025.100228 (PMC12413707; doi:10.1016/j.tjpad.2025.100228)
Supplement: Supplementary file 1 [file mmc1.docx]

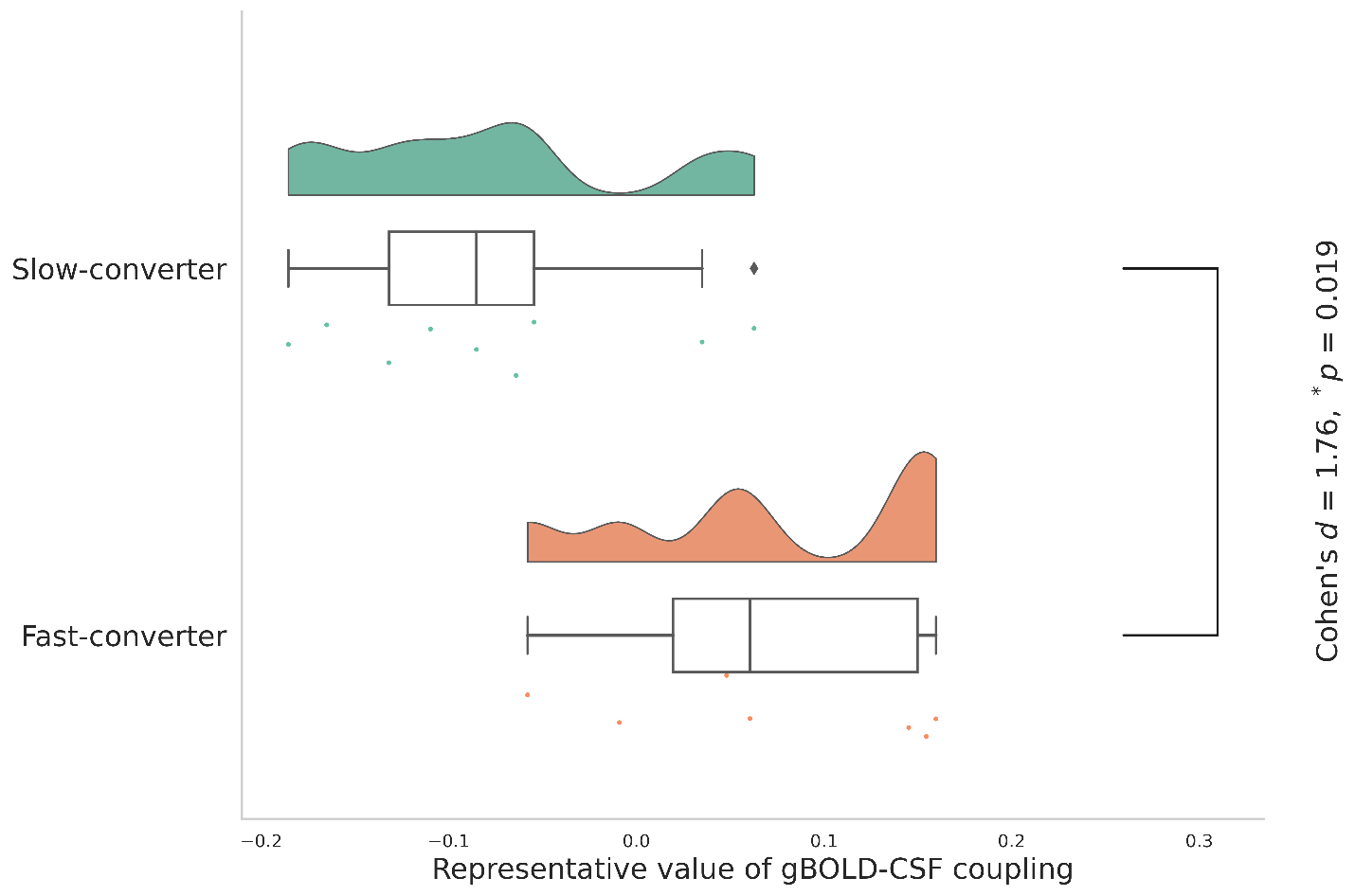


**Supplementary figure 1. Comparison of the gBOLD–CSF coupling between fast-converters and slow-converters**

The representative value of gBOLD–CSF coupling was significantly high in fast-converters compared to slow-converters (Cohen's *d* = 1.76, ^*^*p* = 0.019). Note that the higher the representative value of gBOLD–CSF coupling, the weaker the gBOLD–CSF coupling.

The analysis was performed with adjustments for age, sex, APOE ε4 allele presence/absence, years of education and ADNI phase.

gBOLD, global Blood-Oxygen-Level-Dependent signals; CSF, cerebrospinal fluid.


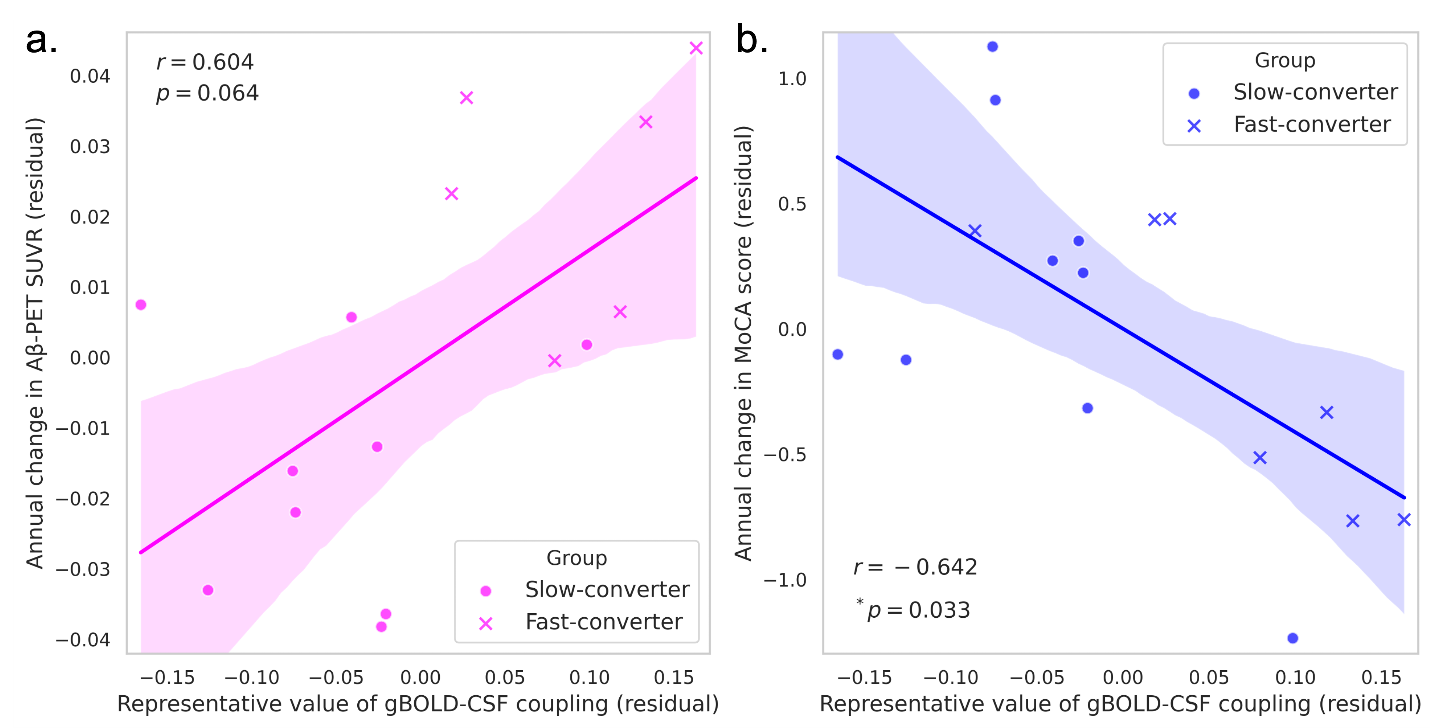


**Supplementary figure 2. Partial correlation between gBOLD–CSF coupling representative value and annual Aβ-PET SUVR and MoCA score change**

a, Partial correlation analysis between the representative value of gBOLD–CSF coupling and the annual change in Aβ-PET SUVR revealed a moderate positive correlation (*r* = 0.604, *p* = 0.064).

b, The partial correlation analysis between the representative value of gBOLD–CSF coupling and the annual change in MoCA score showed a significant strong negative correlation (*r* = −0.642, ^*^*p* = 0.033).

Both plots are adjusted for the following covariates: age, sex, APOE ε4 allele presence/absence, years of education and ADNI phase. The residuals are displayed as separate data points.

Note: Higher representative values of gBOLD–CSF coupling indicate weaker coupling.

Aβ, amyloid beta; PET, positron emission tomography; SUVR, standardized uptake value ratio; MoCA, Montreal Cognitive Assessment.
